# Identification and Characterization of Sebaceous Gland Atrophy-Sparing DGAT1 Inhibitors

**DOI:** 10.1371/journal.pone.0088908

**Published:** 2014-02-18

**Authors:** Eric S. Muise, Yonghua Zhu, Andreas Verras, Bindhu V. Karanam, Judith Gorski, Drew Weingarth, Hua V. Lin, Joyce Hwa, John R. Thompson, Guanghui Hu, Jian Liu, Shuwen He, Robert J. DeVita, Dong-Ming Shen, Shirly Pinto

**Affiliations:** Discovery and Preclinical Sciences, Merck Research Laboratories, Whithouse Station, New Jersey, United States of America; University of Tennessee, United States of America

## Abstract

Inhibition of Diacylglycerol O-acyltransferase 1 (DGAT1) has been a mechanism of interest for metabolic disorders. DGAT1 inhibition has been shown to be a key regulator in an array of metabolic pathways; however, based on the DGAT1 KO mouse phenotype the anticipation is that pharmacological inhibition of DGAT1 could potentially lead to skin related adverse effects. One of the aims in developing small molecule DGAT1 inhibitors that target key metabolic tissues is to avoid activity on skin-localized DGAT1 enzyme. In this report we describe a modeling-based approach to identify molecules with physical properties leading to differential exposure distribution. In addition, we demonstrate histological and RNA based biomarker approaches that can detect sebaceous gland atrophy pre-clinically that could be used as potential biomarkers in a clinical setting.

## Introduction

Diacylglycerol O-acyltransferase 1 (DGAT1) is ubiquitously expressed and catalyzes the final step in triglyceride (TG) synthesis [1]. TG biosynthesis has pleiotropic roles in various tissues. TG can be taken up by the diet and resynthesized in the small intestine by DGAT1 or can be synthesized *de novo* by either DGAT1 or DGAT2 in the liver and/or adipose tissues [2]. Inhibition of DGAT1 in the intestine has been shown to enhance circulating levels of gut incretin levels such as Glucagon-like peptide 1 (GLP-1) and Peptide YY (PYY) post-prandially [3], [4]. In addition to DGAT1's role in these tissues, DGAT1 and DGAT2 have also been demonstrated to be expressed in the skin of mice [5], [6] and human (data not shown). Mice with a deletion of the DGAT1 enzyme (DGAT1 ^-/-^) are protected from diet induced obesity and show increased sensitivities to insulin and leptin and increased energy expenditure [7]. However, in addition to these metabolic phenotypes, DGAT1^-/-^ mice develop leptin-dependent abnormal skin phenotypes, characterized by sebaceous gland atrophy and hair loss [5]. The metabolic effects and the skin phenotype were shown to be recapitulated with pharmacological inhibition of DGAT1 [6]. Skin composition between human and preclinical species varies; wax diester is the major sebum lipid in mouse while TG is the major form in human [8]. Although the exact role of sebum in human is not fully understood, sebum production could be decreased with pharmacological inhibition of skin DGAT1 activity.

Since the identification and the characterization of DGAT1 -/- mice, multiple pharmaceutical companies have been actively pursuing the discovery of small molecule DGAT1 inhibitors to reproduce the beneficial metabolic phenotypes of these mice [9], [10]. Recent early clinical data with DGAT1 inhibitors have uncovered gastrointestinal adverse effects (AEs) as a major issue with no report of adverse skin effects [10]–[12]. However, considering the role of DGAT1 in the skin, such inhibitors represent potential liabilities related to skin AEs as well. To that end one of our goals was to develop small molecule DGAT1 inhibitors with differential exposures at the site of action vs. skin. Low exposures in the skin would protect from skin liabilities while maintaining the beneficial metabolic benefits associated with DGAT1 inhibition in other tissues such as the small intestine. Based on molecular modeling we demonstrated the correlation between lipophilicity of several DGAT1 small molecule inhibitors, skin histological findings and systemic and skin drug exposures. In addition we proposed an RNA-based approach that could be utilized as clinical biomarkers to detect sebaceous gland atrophy driven by DGAT1 inhibitors.

## Results

### Skin effects of DGAT1 inhibitors

**Figure 1:**
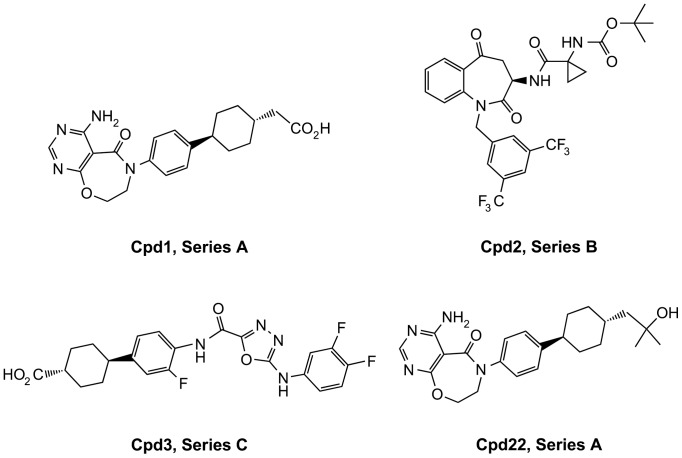
Representative compound structures from three chemical compound series as shown in Table 1.

**Figure 2:**
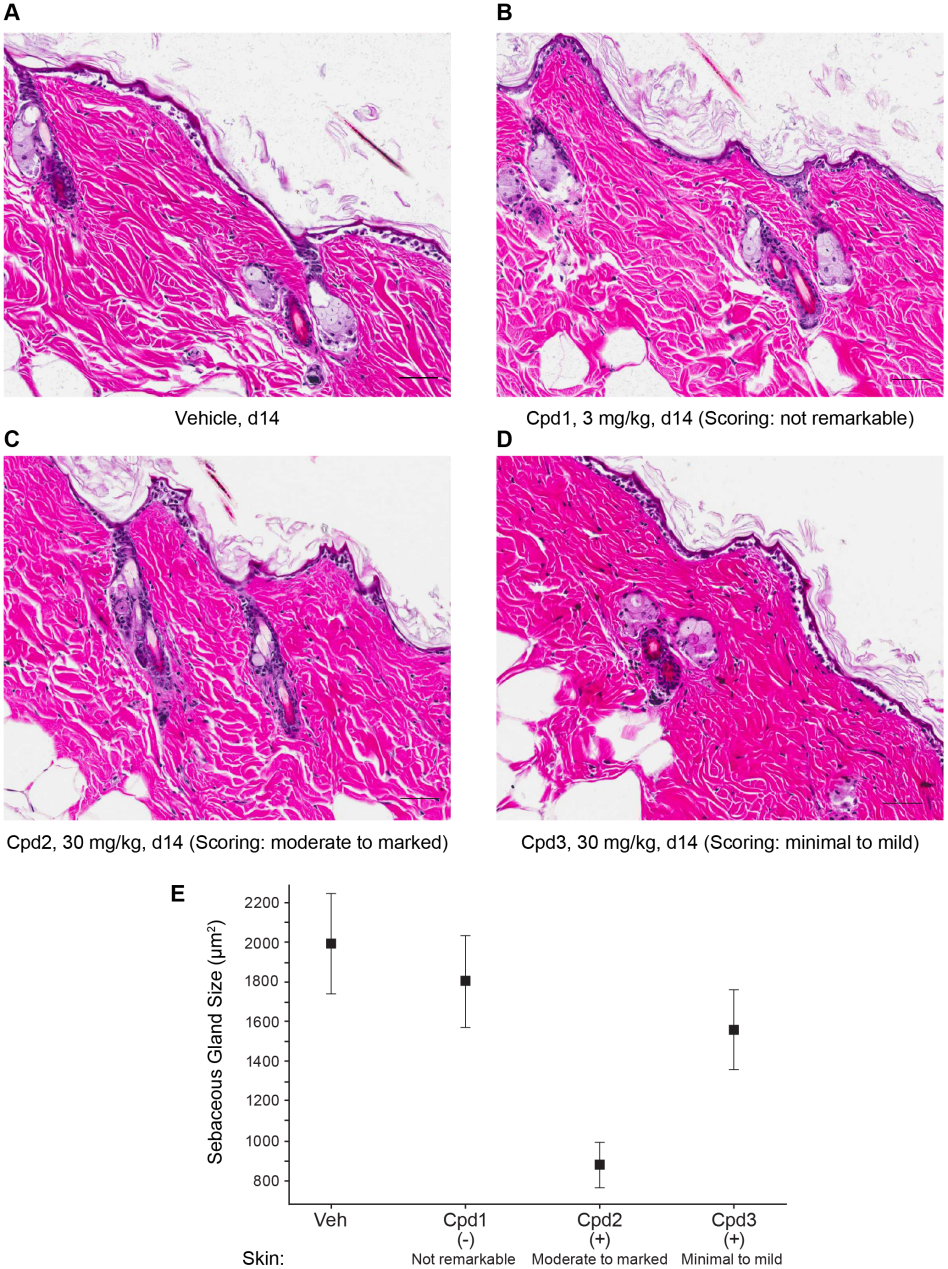
DGAT1 inhibitors with high lipophilicity induce sebaceous gland atrophy. Shown are hematoxylin and eosin stains of dorsal skin biopsies from DIO mice treated with either vehicle (A), Cpd1 (B), Cpd2 (C), or Cpd3 (D) for 14 days at doses indicated. Scoring refers to the histological adverse effect score as described in Table 1. Bar  = 50 µm. The corresponding sebaceous gland sizes (area) are plotted in (E) (and shown in Table S1).

Several DGAT1 inhibitors across different structural classes were tested for their effect on skin morphology after chronic treatment in mice (Figure 1 and Table 1). Compounds were separated into structural classes and assigned to groups A to E. Representative structures from groups A, B, and C are shown in Figure 1 (structures of compounds from groups D and E will be the subject of future reports). After 14 days of oral dosing several compounds either induced sebaceous gland atrophy in the skin or showed no response. As shown in Figure 2, the sebaceous glands in the skin of mice treated with either vehicle or Cpd1 (3 mg/kg, 14 days) appeared normal while the skin of mice treated with Cpd2 (30 mg/kg, 14 days) had moderate to marked atrophic sebaceous glands on the dorsal surface, which were characterized by an overall decreased amount and size of sebaceous gland acini. Skin of mice treated with Cpd3 (30 mg/kg, 14 days) showed minimal to mild effects. The affected sebaceous gland units had fewer acinar cells and/or cells with decreased amount of cytoplasmic vacuolation. Frequently the sebaceous gland acini had consolidated, eosinophilic cytoplasm and pyknotic nuclei. No other histomorphologic changes were observed in these skin sections. Compound plasma exposures (Plasma µM) did not correlate with skin AEs (Scoring) or with skin exposure (Skin/Plasma Ratio; Table 1). However, skin exposures as it related to IC50 did correlate with skin AEs (Skin/IC50). The effects of the compounds were similar on ventral skin (Figure S1). Of note, longer exposure of Cpd1, which did not cause sebaceous gland atrophy at 14 days, in rats at up to 300 mg/kg for 4 weeks had no adverse effects in skin (data not shown). Additional studies will be required to study the effects of these compounds in skin after longer-term exposure.

**Table 1:** Compound characteristics. Cpd1 is the same as DGAT1i in Lin, H et al (2013) [4], while Cpd2 is the same as Compound L in Liu, J et al (2013) [3].

Study	Compound	Series	Plasma µM	Skin µM	Skin/Plasma Ratio	Fu Plasma	mlogD	clogD	mDGAT1 IC50 nM	mDGAT1 IC50 µM	Skin/IC50	Scoring	clogD/mIC50
1	Cpd1	A	0.194	0.0621	0.320	0.310	0.35	0.00	34.6	0.035	1.80	not remarkable	0
**1**	**Cpd2**	**B**	**0.163**	**2.25**	**13.8**	**0.01**	**4.04**	**4.27**	**31.4**	**0.031**	**71.61**	**moderate to marked**	**136**
**1**	**Cpd3**	**C**	**1.38**	**0.135**	**0.098**	**0.063**	**1.73**	**1.83**	**11**	**0.011**	**12.27**	**minimal to mild**	**166**
1	Cpd4	D	0.855	0.111	0.130	0.090	0.90	0.44	58.9	0.059	1.89	not remarkable	7
**2**	**Cpd5**	**D**	**0.461**	**0.138**	**0.298**	**0.09**	**1.08**	**1.61**	**5.2**	**0.005**	**26.45**	**minimal to mild**	**310**
**2**	**Cpd6**	**D**	**2.22**	**0.430**	**0.194**	**0.09**	**1.27**	**1.60**	**18.8**	**0.019**	**22.87**	**moderate to marked**	**85**
2	Cpd7	D	0.171	0.002	0.012	0.098	1.37	0.42	32.4	0.032	0.07	not remarkable	13
**2**	**Cpd2**	**B**	**0.124**	**2.16**	**17.489**	**n/a**	**4.04**	**4.27**	**31**	**0.031**	**69.68**	**moderate to marked**	**138**
2	Cpd8	D	0.0072	0.010	1.345	0.033	1.11	0.48	4.9	0.005	1.96	not remarkable	98
2	Cpd9	D	0.0047	0.006	1.324	0.133	0.94	0.43	12.3	0.012	0.50	not remarkable	35
3	Cpd10	E	0.0782	0.0159	0.203	nd	1.81	0.00	10.9	0.011	1.46	not remarkable	0
3	Cpd11	D	0.199	0.0478	0.241	0.0836	0.73	0.00	29	0.029	1.65	not remarkable	0
**3**	**Cpd12**	**A**	**2.84**	**0.669**	**0.236**	**0.256**	**1.34**	**2.07**	**40.4**	**0.040**	**16.56**	**moderate to marked**	**51**
3	Cpd13	D	0.276	0.0131	0.047	0.06	1.37	0.00	33.3	0.033	0.39	not remarkable	0
3	Cpd14	D	0.209	0.0265	0.127	0.073	1.05	0.00	46.9	0.047	0.57	not remarkable	0
**3**	**Cpd15**	**D**	**1.21**	**0.190**	**0.157**	**nd**	**1.15**	**0.77**	**10.7**	**0.011**	**17.72**	**moderate to marked**	**72**
3	Cpd16	E	0.196	0.0347	0.177	0.059	1.81	0.00	10.4	0.010	3.34	not remarkable	0
**3**	**Cpd2**	**B**	**0.106**	**1.94**	**18.330**	**0.01**	**4.04**	**4.27**	**31**	**0.031**	**62.72**	**moderate to marked**	**138**
**3**	**Cpd6**	**D**	**4.51**	**0.640**	**0.142**	**0.033**	**1.27**	**1.60**	**18.8**	**0.019**	**34.04**	**moderate to marked**	**85**
4	Cpd17	E	1.42	BLQ		0.1021	1.58	0.09	6.3	0.006		not remarkable	14
4	Cpd18	D	1.13	BLQ		nd	0.99	0.65	11.7	0.012		not remarkable	56
4	Cpd19	D	5.38	BLQ		nd	1.20	0.00	17.5	0.018		not remarkable	0
4	Cpd20	D	2.19	BLQ		0.0495	0.99	0.35	34.2	0.034		not remarkable	10
4	Cpd21	D	8.01	BLQ		nd	1.04	0.11	17.2	0.017		not remarkable	6
**4**	**Cpd22**	**A**	**51.4**	**BLQ**		**0.112**	**2.15**	**2.87**	**25.9**	**0.026**		**moderate to marked**	**111**
**4**	**Cpd6**	**D**	**8.39**	**BLQ**		**0.033**	**1.27**	**1.60**	**18.8**	**0.019**		**moderate to marked**	**85**

Plasma  =  plasma concentration; Skin  =  skin concentration; Skin/Plasma Ratio  =  skin to plasma concentration ratio; Fu Plasma  =  unbound fraction in plasma; mlogD  =  measured logD; clogD  =  calculated logD using ACD (measure of lipophilicity); mDGAT1 IC50  =  in vitro potency; Skin/IC50 =  skin concentration vs in vitro potency; Scoring  =  histological adverse effect score; clogD/mIC50 =  calculated logD vs in vitro potency; BLQ  =  below detection. Bold  =  compounds causing atrophy.

### Plasma and Skin Concentrations of Compounds

Plasma and corresponding skin concentrations of the compounds are shown in Table 1. As can be seen, following dosing of the compounds at 30 mg/kg for 14 days, the relative trough levels of the compounds in plasma and skin do not have a direct correlation. Compounds that exhibit high plasma exposures do not appear to have high skin exposure and vice versa. Similar lack of correlation is also observed with unbound plasma concentrations and skin exposures.

### Lipophilicity of the Compounds

**Figure 3:**
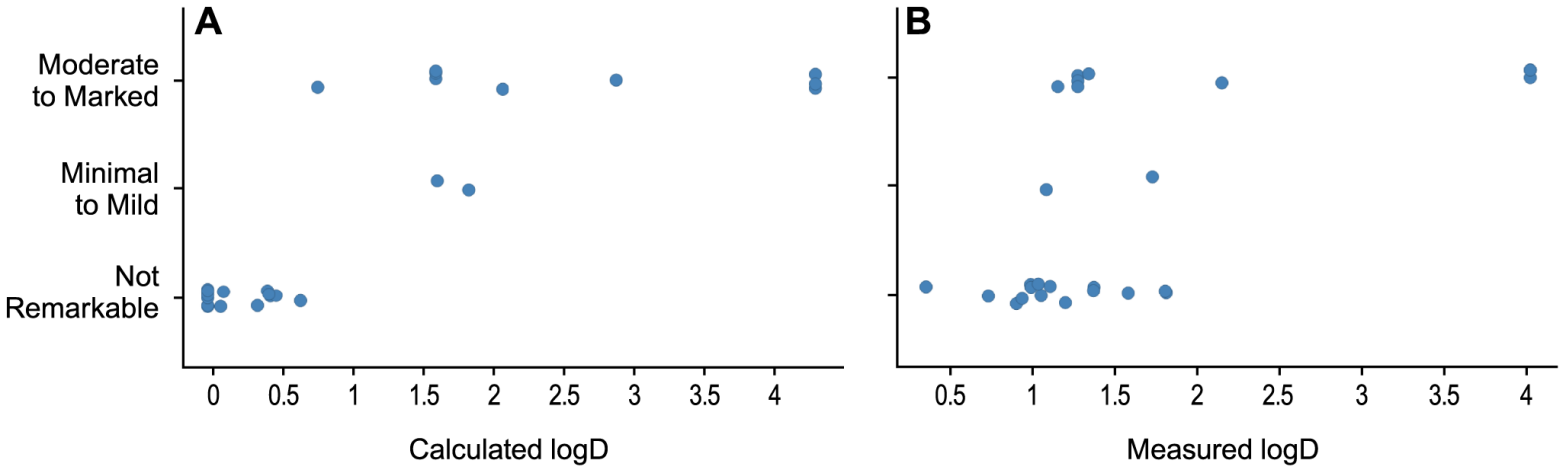
Histopathology versus lipophilicity. Calculated logD (clogD, A) or measured logD (mlogD, B) versus observed skin adverse effects. Data is jittered on the y-axis to improve visualization.

To gain a better understanding of the chemical physical properties of DGAT1 inhibitors as it relates to skin AEs, we evaluated lipophlicity (Log D) of these compounds which can be either measured by HPLC (mlogD) or calculated using the ACD software (clogD). Distribution into skin can be understood as a partitioning equilibrium dependent on compound properties. As listed in Table 1, 7 out of 22 compounds tested (Cpd2, 3, 5, 6, 12, 15, 22) were found to induce either “minimal to mild” or “moderate to marked” skin sebaceous gland atrophy after 14 days of treatment. Compounds with sufficient potency against the mouse enzyme (mDGAT1 IC50 nM) only show skin toxicity as measured by histopathology at sufficient exposure levels in the skin (Table 1). Interestingly, we find that compounds with skin effects can be distinguished from compounds without observable skin effects by plotting lipophilicity (clogD) against the observed histopathology (Figure 3). Compounds which show significant or minimal adverse effects in skin generally have a predicted clogD of greater than 1, while compounds with no observable effects all have a predicted clogD of less than 0.7.

We also plotted mlogD versus observed histopathology and found considerable overlap between the compounds showing and lacking adverse skin effects (Figure 3). While the mlogD and clogD are correlated, there is a poorer correlation of the most polar compounds and the mlogD does not correctly predict histopathological findings.

**Figure 4:**
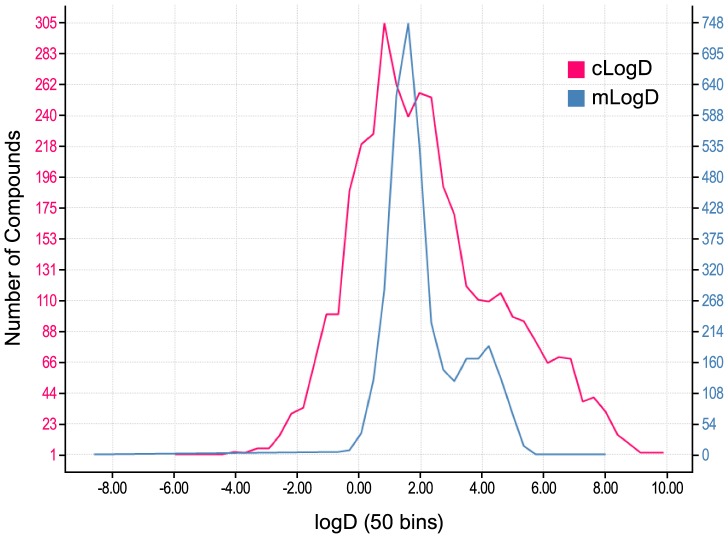
Distribution of logD values by method. The frequency of logD values of DGAT1 compounds for mlogD (blue) and clogD (red).

To better understand why measured logD more poorly differentiated compounds than calculated logD we plotted the frequency of logD measurements for about 4,000 DGAT1 compounds by method (Figure 4). While the two logD methods were correlated (R^2^ 0.76), we found that the dynamic range of measured logD was significantly less than the calculated range with values ranging from 0 to 5.5, while calculated logD measurements ranged from −3 to 9. The shoulder at mlogD five represents the more lipophilic series B compounds and is less pronounced, but visible, in the clogD distribution. The narrower range of HPLC measured logD measurements does not significantly distinguish compounds, while the ACD calculated logD values allow for more differentiation.

### RNA profiling of skin biopsies

**Figure 5:**
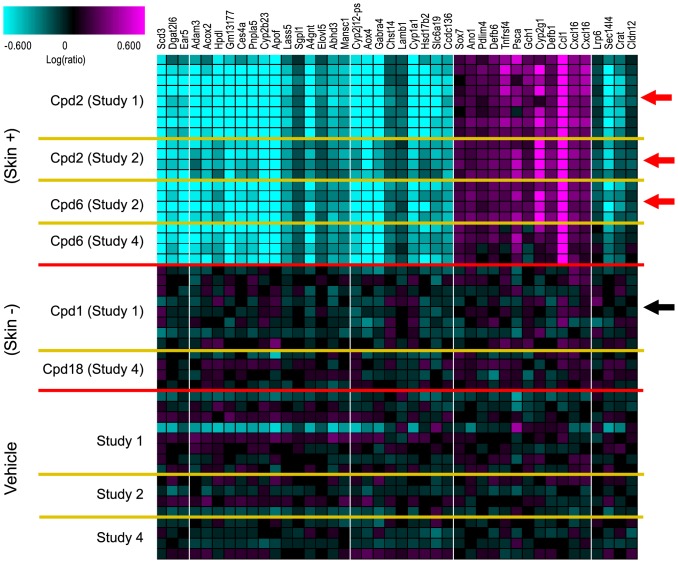
RNA biomarkers for sebaceous gland atrophy in skin. Shown are the 42 probesets, identified in the Training Set (Studies 1 and 2), that were regulated by skin-positive compound treatments (those that produced sebaceous gland atrophy) but not by the skin-negative compound treatments (the one that did *not* produce sebaceous gland atrophy). After excluding the absent probes (low intensity), these 42 probesets met the following cutoffs: 1.2 fold change and ANOVA p<0.01 between all 3 skin-positive compound treatments (red arrows) and their respective vehicle treatments, and ANOVA p>0.1 between the skin negative compound treatment (black arrow) and its respective vehicle treatment. The probesets for RIKEN genes were excluded. Plotted are the LogRatio values (+/− 4 fold fold scale) with magenta representing up-regulated probesets and cyan representing down-regulated probesets. Treatments from the independent Test Set (Study 4) are included for comparison but were not used to identify the 42 probesets.

**Figure 6:**
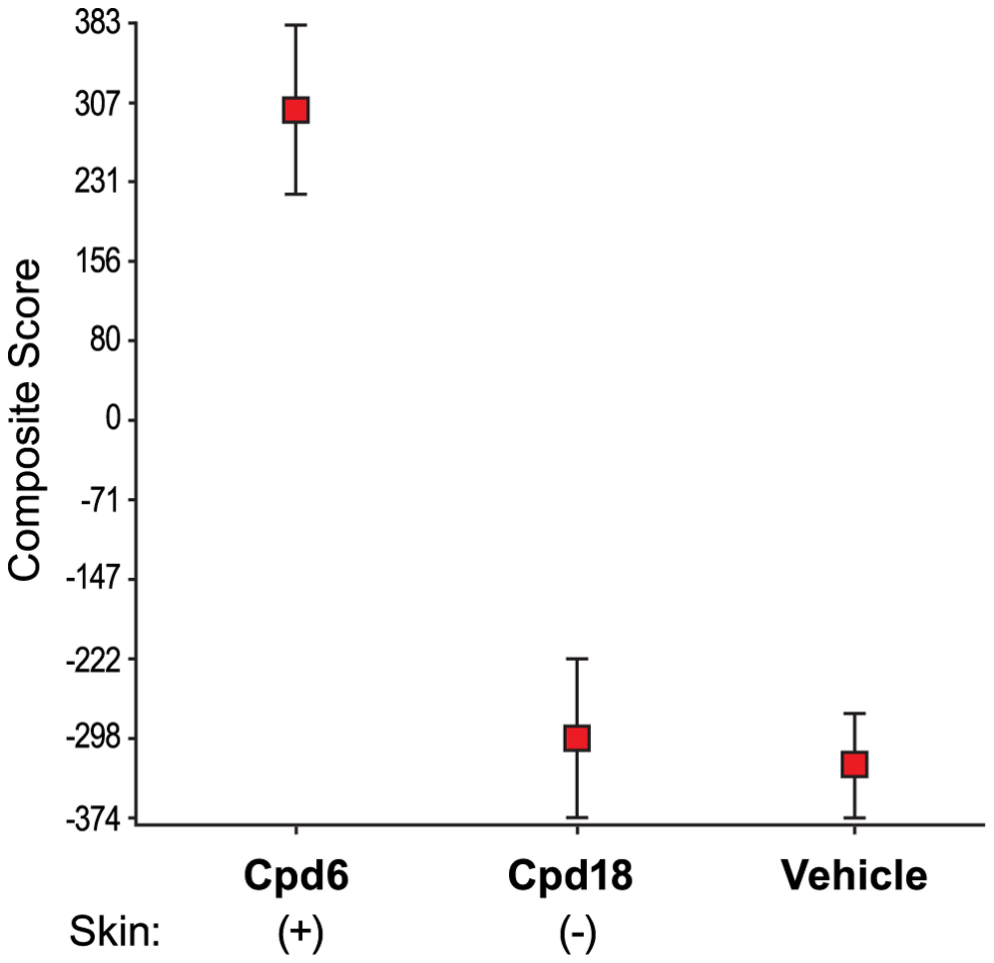
Validation of the 42 probeset-composite score in an independent Test Set. The 42 probesets from the Training Set, shown in Figure 5, were used to generate a composite score across the treatments from the independent Test Set (Study 4). The p value between Cpd6 treatment (skin-positive) and Cpd18 treatment (skin-negative), in Study 4, is less than 0.0001. Expression data from this set of RNA biomarkers is predictive for sebaceous gland atrophy in mice following DGAT inhibitor treatment.

**Figure 7:**
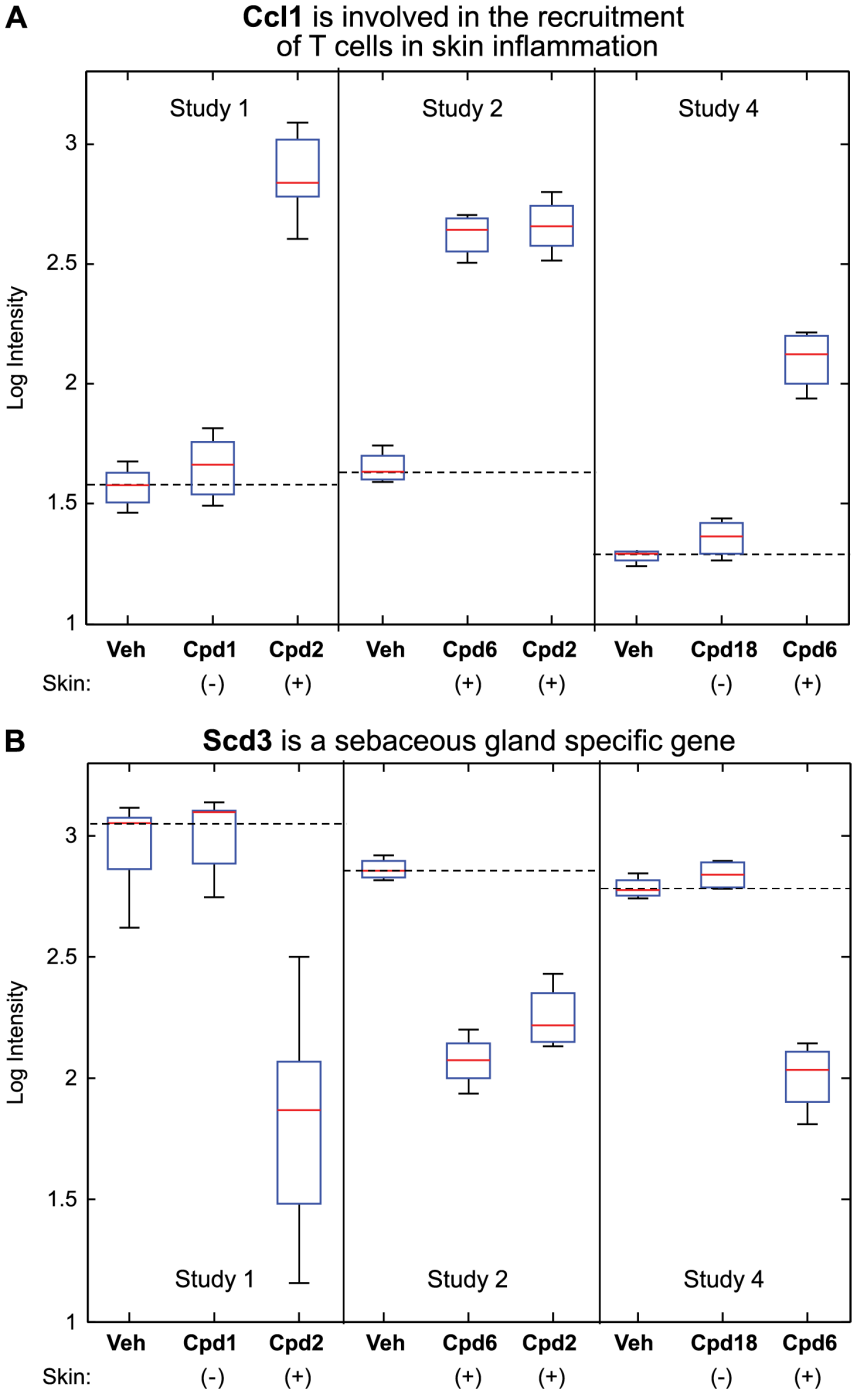
Immune-regulated genes are up-regulated, while lipid metabolism genes are down-regulated, with sebaceous gland atrophy. Box plots of probesets regulated by the skin-positive DGAT1 inhibitors (those that produce sebaceous gland atrophy) but not by the skin-negative compounds (those that do *not* produce sebaceous gland atrophy). Plotted are the LogIntensity values across the replicates in each group, and across the three studies. Ccl1 (A; chemokine (C-C motif) ligand 1) is involved in the recruitment of T cells in skin inflammation; and Scd3 (B; stearoyl-coenzyme A desaturase 3) is a sebaceous gland specific gene.

To identify biomarkers underlying the skin pathophysiology associated with DGAT1 inhibition we initiated global gene expression profiling. Diet induced obese (DIO) mice were treated with several structurally diverse DGAT1 inhibitors for 14 days and total RNA from skin biopsies were profiled on Affymetrix custom microarrays. Forty two probesets were identified using a Training Set consisting of two skin-positive compound treatments (compounds inducing sebaceous gland atrophy) and one skin-negative compound treatment (compound *not* inducing sebaceous gland atrophy; Figure 5 and Table 2). A composite score using these 42 probesets and an independent Test Set (treatments not used to identify the biomarkers) was able to differentiate between the skin-positive and the skin-negative compound treatments with p<0.0001 (Figure 6). Up-regulated genes in this set include proteins involved in the immune response such as Ccl1 (Chemokine (C-C motif) ligand 1), Defb1 (Defensin beta 1) and Cxcl16 (Chemokine (C-X-C motif) ligand 16) (Figure 7A and Table 2). Down-regulated genes include proteins involved in lipid, fatty acid, and steroid metabolism such as Scd3 (Stearoyl-coenzyme A desaturase 3), Acox2 (Acyl-Coenzyme A oxidase 2, branched chain), and Elovl5 (ELOVL family member 5) (Figure 7B and Table 2) consistent with the DGAT1 pathway. Twenty six of these 42 probesets were significantly regulated by the skin-positive compound in the Test Set and not by the skin-negative compound treatment (Table 2).

**Table 2:** RNA biomarkers for sebaceous gland atrophy in skin. Listed are the 41 unique genes from the 42 probesets identified in the Training Set as shown in Figure 4 (Cxcl16 had 2 probesets).

		Cpd2 vs Vehicle		Cpd2 vs Vehicle		Cpd6 vs Vehicle		Cpd6 vs Vehicle		Cpd1 vs Vehicle		Cpd18 vs Vehicle	
		Study 1-Training		Study 2-Training		Study 2-Training		Study 4-Test		Study 1-Training		Study 4-Test	
		Skin-Positive		Skin-Positive		Skin-Positive		Skin-Positive		Skin-Negative		Skin-Negative	
Gene Symbol	Entrez ID	Fold Change	p	Fold Change	p	Fold Change	p	Fold Change	p	Fold Change	p	Fold Change	p
**Ccl1**	20290	19.9	**	10.2	**	9.3	**	6.6	**	1.2		1.2	
Cyp2g1	13108	2.4	**	5.7	**	3.8	**	1.5		1.1		1.0	
Psca	72373	1.9	**	2.2	**	1.9	**	1.8	*	−1.0		1.1	
Cxcl16	66102	2.0	**	1.8	**	1.8	**	1.5	*	1.1		1.2	
Defb6	116746	1.5	**	1.6	**	1.6	**	1.3	*	1.1		−1.1	
Cxcl16	66102	2.1	**	1.7	**	1.6	**	1.2	*	1.1		1.2	*
Tnfrsf4	22163	2.1	**	1.6	**	1.5	**	1.0		1.1		1.1	
Defb1	13214	1.7	**	1.7	**	1.7	**	1.3	*	−1.1		−1.0	
Ano1	101772	1.5	**	1.4	**	1.6	**	1.3	**	1.1		1.1	**
Gch1	14528	1.6	**	1.3	**	1.4	**	−1.2		1.1		−1.2	
Sox7	20680	1.2	**	1.5	**	1.4	**	1.5	**	−1.1		1.2	*
Pdlim4	30794	1.3	**	1.5	**	1.3	**	1.1		1.0		1.0	
Cldn12	64945	−1.2	**	−1.3	**	−1.3	**	1.0		1.0		1.0	
Lrp6	16974	−1.2	**	−1.3	**	−1.4	**	1.1		1.0		1.0	
**Sgpl1**	20397	−1.4	**	−1.3	**	−1.4	**	−1.4	**	1.0		1.0	
Lamb1	16777	−1.5	**	−1.3	**	−1.5	**	−1.2		1.0		−1.1	
**Chst14**	72136	−1.6	**	−1.4	**	−1.8	**	−1.6	**	1.1		1.0	
**Elovl5**	68801	−1.7	**	−1.4	**	−1.6	**	−1.5	**	1.0		1.0	
Crat	12908	−2.2	**	−1.4	**	−1.9	**	−1.3	*	1.1		1.1	
**Mansc1**	67729	−2.2	**	−1.8	**	−1.8	**	−1.9	**	−1.0		1.0	
**Abhd3**	106861	−2.4	**	−1.7	**	−2.1	**	−2.1	**	−1.1		−1.0	
**Lass5**	71949	−2.4	**	−2.1	**	−2.2	**	−2.2	**	−1.1		−1.1	
**Ccdc136**	232664	−2.6	**	−2.3	**	−2.3	**	−2.1	**	−1.1		1.1	
Slc6a19	74338	−3.1	**	−1.9	**	−2.2	**	−2.1	*	−1.2		−1.1	
**Sec14l4**	103655	−3.3	**	−2.4	**	−3.0	**	−1.8	**	−1.0		1.2	
**Hpdl**	242642	−4.1	**	−2.0	**	−2.5	**	−2.6	**	1.1		1.0	
**Hsd17b2**	15486	−4.2	**	−1.7	**	−2.6	**	−2.7	**	−1.2		−1.0	
**Acox2**	93732	−3.4	**	−2.6	**	−3.4	**	−3.1	**	1.0		1.2	
**Dgat2l6**	668257	−3.3	**	−2.9	**	−3.3	**	−3.0	**	−1.0		1.0	
**Adam3**	11497	−5.0	**	−2.1	**	−3.6	**	−3.6	**	−1.1		−1.1	
**Cyp1a1**	13076	−5.4	**	−2.5	**	−4.0	**	−2.2	**	1.2		−1.1	
**A4gnt**	333424	−5.3	**	−2.0	**	−3.9	**	−3.4	**	−1.1		1.0	
**Cyp2j12-ps**	242546	−5.0	**	−2.1	**	−4.7	**	−3.0	**	−1.1		1.2	
**Ces4a**	234677	−5.2	**	−2.6	**	−4.2	**	−3.1	**	1.0		1.1	
**Pnpla5**	75772	−5.7	**	−2.5	**	−3.9	**	−3.2	**	1.0		−1.0	
**Ear5**	54159	−3.5	**	−3.4	**	−5.3	**	−5.7	**	−1.1		1.0	
**Gabra4**	14397	−6.4	**	−2.1	**	−5.6	**	−3.8	**	−1.2		−1.0	
**Gm13177**	435815	−6.2	**	−4.1	**	−7.6	**	−4.7	**	1.0		1.0	
**Aox4**	71872	−7.9	**	−4.1	**	−7.6	**	−6.3	**	−1.2		−1.0	
**Scd3**	30049	−14.1	**	−4.1	**	−6.2	**	−6.0	**	1.1		1.1	
**Apof**	103161	−9.6	**	−5.2	**	−6.9	**	−8.2	**	1.3		−1.2	
**Cyp2b23**	243881	−13.7	**	−14.2	**	−17.2	**	−10.7	**	1.1		−1.0	

Fold change and ANOVA p values for compound treatments compared to their respective vehicle treatments, for both the Training and Test Sets, are included. The 26 probesets that are also significantly regulated in the Test Set are shown in bold. **  = ANOVA p<0.01; *  = ANOVA p<0.05; $ = ANOVA p<0.1.

## Discussion

Potent DGAT1 inhibitors are currently being developed for the treatment of hyper-triglyceridemia and obesity [6]. The major adverse effect observed so far in the clinic relates to gastrointestinal effects (nausea, diarrhea, and vomiting), with no report of skin issues [10]. Nevertheless, to avoid potential skin AEs, especially after chronic treatment, effective therapeutics will selectively inhibit DGAT1 in the liver and gut with minimal activity on other tissues such as skin. We described compound lipophilicity as a predictor of skin exposure with subsequent induction of sebaceous gland atrophy. The distribution coefficient, D, is a pH dependent measure of the propensity of a molecule to differentially dissolve in two immiscible phases, taking into account all ionized and unionized forms (microspecies). It serves as a quantitative descriptor of lipophilicity.

Interestingly, many compounds which carry a carboxylic acid moiety are associated with a lack of skin AEs (Cpd1 is an example). It is likely that the carboxylic acid leads to decreased lipophilicity which prevents the compound from entering the skin. To address this hypothesis, we modified Cpd1 and replaced the carboxylic acid with a tertiary alcohol group (Cpd22). As predicted, this compound led to a moderate to marked skin histological score with high skin compound exposure levels. Intriguingly Cpd6 possesses a similar carboxylic acid group (not shown) but scored moderate to marked for skin AEs. The calculated clogD of Cpd6 is significantly higher (1.60) than other compounds that possess the carboxylic group, and which do not induce skin AEs, thus illustrating that molecular physical properties, and not functional features, are a better predictor of adverse skin effects. Consistent with association of reduced clogD, skin exposure/IC50 were significantly lower in compounds that did not lead to morphological skin changes.

The compound treatment-induced sebaceous gland atrophy could be detected histologically, after 14 days of treatment. This technique was labor and time intensive thus we performed a microarray study to identify potential markers that could report on this skin effect and that could be potentially developed into a robust higher throughput qPCR assay. Forty two probesets were identified that were regulated by the sebaceous gland atrophy-inducing DGAT1 inhibitors. Several genes involved in the immune response were up-regulated. In fact, Ccl1 was the most robustly up-regulated gene by the DGAT1 inhibitors that caused sebaceous gland atrophy and it has been reported to be increased in atopic skin inflammation [13]. This could be a common marker of skin inflammation.

In contrast, genes involved in lipid and steroid metabolism were down-regulated consistent with inhibition of the DGAT1 pathway. Of these, Scd3 and Aox4 (Aldehyde oxidase 4) were some of the most robust. They are expressed in mouse skin and in particular sebaceous glands [14], [15]. Scd3 is involved in the conversion of saturated fatty acids into monounsaturated fatty acids [14], while Aox4 is involved with local synthesis and bio-disposition of endogenous retinoids [15]. Hsd17b2 (Hydroxysteroid (17-beta) dehydrogenase 2) is expressed in human sebaceous glands and has been shown to be important in regulating the hormonal milieu by interconverting weak and potent androgens and estrogens in these glands [16]. The down-regulation of these lipid and retinoid metabolizing genes is in line with atrophy of the sebaceous glands. Identification of molecular markers of these skin adverse effects could prove useful in the development of skin-sparing DGAT1 inhibitors.

One of the challenges associated with identification of DGAT1 small molecule inhibitors was to identify potent efficacious molecules devoid of skin issues which were predicted from the DGAT1^-/-^ mouse model. The identification of an association between compound lipophilicity and skin adverse effects was instrumental in advancing the drug discovery effort for the identification of DGAT1 small molecule inhibitors with minimal impact in the skin. Furthermore molecular markers in mouse skin were identified that could potentially serve as early readouts of adverse events in a clinical setting. It will be important however to determine if these findings from the murine system are also relevant in human skin as there are known differences in sebaceous gland biology/pathology across species. In that sense, markers already associated with sebaceous gland function or inflammation in humans (Hsd17b2 and Ccl1) might be the most promising candidates for clinical markers of sebaceous gland atrophy.

## Methods

### Ethics Statement

All animal procedures were reviewed and approved by the Institutional Animal Care and Use Committee of Merck & Co., Inc.

### Compounds

All compounds were provided by Merck Research Laboratories (Rahway, NJ). Cpd2 in this report is compound L in [3].

### In Vivo Treatments

Experiments were performed in male diet induced obese C57BL/6 mice (Taconic Farms, Germantown, NY) that were fed high-fat diet (D12492: 60% kcal from fat; Research Diets) in a 12 hr light/12 hr dark cycle. Animal protocols used in these studies were approved by the Merck Research Laboratories Institutional Animal Care and Use Committee (Rahway, NJ). Mice were acclimated to nonspecific stress for 7 days before the onset of the chronic dosing studies where they were dosed orally with vehicle (0.5% methylcellulose) or compounds at 30 mg/kg, or at doses indicated, for 14 days.

### Histology

Skin samples from DIO mice treated with DGAT1 inhibitors for 14 days were collected from the dorsal and/or ventral surface and immediately fixed in 10% buffered formalin for 24 hr at room temperature and embedded in paraffin. Paraffin specimens were sectioned at 5 µm and stained with hematoxylin and eosin, and were evaluated blindly with light microscopy. In general, the severity scores were determined using the following criteria: “not remarkable”  =  no abnormalities; “minimal”, “mild”, “moderate”, “marked”, and “severe”  =  less than 10%, 10–25%, 25–50%, 50–75%, and greater than 75% of sebaceous gland units with some evidence of atrophy, respectively. One slide/location with at least 50 sebaceous glands was used for scoring purposes. Digital images were captured using a 20× objective and the ScanScope XT slide scanner system (Aperio Technologies, Vista, CA) and managed using Spectrum (Aperio). The size of the sebaceous glands were measured using ImageScope of Aperio system by the method previously described [17].

### Plasma Compound Concentration

Concentrations of compounds in plasma were determined by LC-MS/MS following protein precipitation. To 100 µL of plasma sample, 20 µL of an acetonitrile solution of the internal standard (2,500 ng/ml), was added. This step was followed by protein precipitation with 300 µL of acetonitrile. Samples expected to contain analyte concentrations exceeding the upper limits of the calibration curves were diluted with control plasma. Sample mixtures were vortex-mixed for 15 minutes. After spinning in a centrifuge for 15 minutes at 1,000× g (at 10°C), the supernatants were transferred by a Tomtec Quadra 96 Model 320 (Hamden, CT) to Empore 96 filter plates (Fisher Scientific, Springfield, NJ), and the resulting filtrates collected. These filtrates were dried at 25°C under a stream of nitrogen gas, on a Zymark TurboVap 96 (Hopkinton, MA). The residues were reconstituted with 100 µL of a 8∶2, v/v, mixture of mobile phases A (20 mM aqueous ammonium acetate containing 0.01% formic acid), and B (80∶20, v/v, acetonitrile:methanol), vortex-mixed, spun in a centrifuge at 1,000× g (at 10°C), then analyzed by LC-MS/MS.

The LC-MS/MS system comprised of a Leap Technologies HTS-PAL autosampler (Carrboro, North Carolina), two Perkin Elmer Series 200 Micro Pumps (Norwalk, CT), an in-line pre-column filter, a Type W 6-port Valco switching valve (Vici, Houston, TX), and a Sciex API 365 mass spectrometer (Toronto, Canada). Chromatographic separation of the analytes was achieved by RP-HPLC.

Data were acquired and processed by Sciex Analyst version 1.1 software. Peak area ratios of analyte to internal standard were plotted as a function of the nominal concentrations of the analyte. A line of best fit was generated from the curve points by linear regression with a weighting factor of 1/x2. The equation of this line was then used to calculate the plasma ratios in samples.

### Skin Compound Concentration

The skin tissue samples from mice were weighted and placed in 48-well plates (or tubes). A volume of water equivalent to three times the skin weight was added to each tube and homogenized by a Tissue Terror (Biospec Products, Inc.; Model 985-370). An aliquot of 100 µL homogenized blank skin tissue samples were transferred to 96-well plates designated for standard curve and QCs. Standard curve and QCs were prepared by adding aliquots of 25 µL working standard solutions (1–8000 ng/mL), to generate a tissue calibration curve of 1–8000 ng/mL. An aliquot of 100 µL dosed skin tissue homogenates were transferred to the 96-well plate and a 25 µL aliquot of acetonitrile:water 1∶1 was added into each well containing dosed tissue samples to make the volumes of all samples, stds, and QCs equal. Protein precipitation was used for sample extraction by the addition of 500 µL of acetonitrile crash solution, containing IS. Internal standard used was Alprazolam (Sigma Aldrich) at 100 µM in the crash solution. Samples were vortexed for 30 seconds and then centrifuged at 4000 rpm for 5 min. The supernatant was transferred to a separate plate.

A Thermo Scientific-Cohesive LX-2 system consisting of a CTC Analytics autosampler, Flux Instruments AG pumps, and a valve module, controlled by Aria software was used. A Sciex API-4000 mass spectrometer (Toronto, Canada) was the detector using Analyst 1.4.2 software. The aqueous mobile phase (A) was water with 0.1% formic acid and the organic mobile phase (B) was acetonitrile with 0.1% formic acid. A gradient chromatographic profile was used. An initial condition of 95% A and 5% B was held for 15 seconds. Then over 90 seconds, the chromatic conditions were ramped to 5% A and 95% B. This was held for 25 seconds when the conditions were changed back to original 95% A and 5% B and held for 50 seconds. The flow rate was constant at 0.75 mL/min and a Waters Acquity UPLC HSS T3 1.8 µM, 2.1 mm×50 mm column (Waters, 186003538) was used. A turbo ionspray interface was used in positive ion mode for detection of analytes. Multiple reaction monitoring (MRM) was used to measure the analyte response. The peak area ratio of analyte to internal standard for nominal concentrations was used to make a linear regression line with a 1/x2 weighting factor. The line's equation was used to determine the concentration in the unknown samples.

### Unbound Fraction (Fu) Determination

Plasma protein binding was determined by equilibrium dialysis with an incubation period of 4 hr at 37°C. The dialysis membrane (10-12K MWCO, HTDialysis, LLC) was hydrated and the dialysis apparatus (HTDialysis, LLC) assembled according to the manufacturer's directions. A 150 µL aliquot of mouse plasma containing compound (5 µM) was added to one side of the dialysis membrane. A 150 µL aliquot of phosphate buffered saline (pH 7.4) was added to the opposite side. The plate was then sealed and placed in a shaking incubator set to maintain a temperature of 37°C and constant shaking rate of 60 rpm for 4 hr. At the end of the incubation period, 50 µL aliquots of plasma and buffer were removed and analyzed by LC-MS/MS. Percent unbound was determined as the amount of compound in buffer (post incubation) divided by amount of compound in plasma (post incubation) x100.

### Measured logD

LogD was measured by high-throughput (HT) HPLC at pH 7.0. The chromatographic system consisted of an Agilent 1200 HPLC system composed of a G1379B degasser, G1312B binary pump, G1367C well-plate autosampler, G1316B thermostatted column compartment, G1315C diode-array UV-vis detector, and ChemStation software, all from Agilent Technologies, USA. The separations were carried out on a Poroshell 120 EC-C18, 30 mm×3.0 mm I.D., 2.7 µm, (Agilent Technologies, USA). The mobile phase consisted of phosphate buffered saline at pH 7 (mobile phase A) and acetonitrile (mobile phase B). The column oven temperature was set to 30°C. The HPLC analysis began with an isocratic step of 0.2 minutes at 5% B at 1.5 ml/min, followed by a gradient from 5% to 98% B in 1.0 minute at 1.5 ml/min. A second isocratic step of 0.2 minutes at 98% B with a changing flow rate from 1.5 to 2.0 ml/min was then followed by a gradient from 98 to 5% B in 0.1 minutes with the flow rate changing from 2.0 to 1.5 ml/min. The equilibration time between injections is 0.2 minutes at 5% B. The injection volume is 5 µL and the spectrophotometric detection was set to 215, 238 and 254 nm.

A 10 mM DMSO stock solution of compound was delivered for analysis. A 100 µM standard solution was generated by diluting 10 µL of the 10 mM stock solution with 990 µL of diluent (10% DMSO/10% MeCN/80% MeOH, v/v/v). The chromatographic system was calibrated with a set of standards with published shake-flask logD values. Linear regression was used to determine the calibration line relating the retention time to logD for the calibration standards. This line was then used to determine the HT HPLC logD7.0 value of compound from the measured retention time by the HPLC/DAD analysis of the 100 µM standard solution.

### Calculated logD

LogD was calculated using the ACD Labs v10 LogD Predictor software at pH 7.4 for all compounds (ACD Labs, Toronto, Canada)[18].

### DGAT1 Enzyme Assay

The activity of DGAT1 inhibition was measured using membrane preparations from Pichia overexpressing human DGAT1 and DG/oleoyl CoA as substrates at Km concentrations in the presence of CPM (Life Technologies, D346), which is weakly fluorescent until reacted with free thiols of CoA released from oleoyl CoA after it is incorporated into diacylglycerol to form TG. Mock membranes showed minimal activity (data not shown). IC50s were calculated using either Assay Data Analyzer or GraphPad Prism4 software [3].

### RNA Profiling

Skin biopsies (3–5 mm^2^) from dorsal and/or ventral surfaces from DIO mice treated with compounds for 14 days were obtained and placed in RNAlater solution (Ambion, Austin, TX) at 4°C, according to the manufacturer's instructions. After removal from the RNAlater solution, the samples underwent a freeze/manual pulverization step prior to Trizol (Invitrogen, Carlsbad, CA) polytron chloroform extraction. RNA was isolated using the Promega SV-96 total RNA kit (Promega, Madison, WI) according to the manufacturer's instructions. Samples were amplified and labeled using a custom automated version of the NuGEN Ovation WB protocol. Hybridization on custom mouse Affymetrix microarrays (Santa Clara, CA), labeling and scanning using Affymetrix ovens, fluidics stations and scanners were performed according to the protocols recommended (NuGEN, San Carlos, CA). Sample amplification, labeling, and microarray processing were performed by the Covance Genomics Laboratory in Seattle, WA. The raw gene expression data has been deposited into the Gene Expression Omnibus database (http://www.ncbi.nlm.nih.gov/geo/query/acc.cgi?acc=GSE49375).

One-way ANOVA analysis was performed with Matlab (The Mathworks, Natick, MA). Probesets had to pass a pre-filter of Affymetrix MAS5 present call p-value <0.05 in more than 50% of the samples to qualify for further analysis. Average fold changes between compound and vehicle treatment groups were generated with the vehicle as baseline. Differentially expressed probesets (signature genes) were selected with ANOVA p value <0.01 and average fold change greater than 1.2 fold (up- and down-regulated).

42 probesets that correlated with sebaceous gland atrophy in the skin were identified from a training set of two skin-positive compound treatments (Cpd2 and Cpd6, producing sebaceous gland atrophy) and one skin-negative compound treatment (Cpd1, *not* producing sebaceous gland atrophy) and spanning two independent studies. After excluding the absent probesets (low intensity), the 42 probesets were identified that met the following cutoffs: 1.2 fold change and 1-way ANOVA p<0.01 between all 3 skin-positive compound treatments (Cpd2 was profiled in each of the two studies) and their respective vehicle treatments. The probesets that had a 1-way ANOVA p<0.1 between the skin-negative compound treatment and its respective vehicle treatment were removed (as well as unknown RIKEN genes).

A composite score was computed using these 42 probesets for the treatments in the independent Test Set (Cpd6, skin-positive and Cpd18, skin-negative treatments). The score was calculated by subtracting the averaged expression value of the down-regulated 29 probesets from the averaged expression value of the up-regulated 13 probesets as follows:
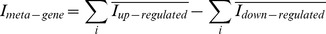



The significance p value of the t-test was performed on the composite scores between the two treatment groups, and their averaged scores were calculated as least-square means.

Of the 42 probesets identified in the Training Set, 26 were also significantly regulated in the Test Set by the skin-positive compound (Cpd6; 1.2 fold change and ANOVA p<0.01) and not by the skin-negative compound (Cpd18; ANOVA p>0.1).

## Supporting Information

Figure S1 **DGAT1 inhibitors with high lipophilicity induce sebaceous gland atrophy.** Shown are hematoxylin and eosin stains of ventral skin biopsies from DIO mice treated with either vehicle (A), Cpd1 (B), or Cpd2 (C) for 14 days at doses indicated. Scoring refers to the histological adverse effect score as described in Table 1. Bar  = 50 µm.
(PDF)

Table S1 **DGAT1 inhibitors with high lipophilicity induce sebaceous gland atrophy.** Shown are the sebaceous gland sizes from dorsal skin biopsies from DIO mice treated with vehicle, Cpd1, Cpd2, or Cpd3 for 14 days. The data are plotted in Figure 2E.
(XLSX)
