# Supplementary figures and images for: Identification and Characterization of Sebaceous Gland Atrophy-Sparing DGAT1 Inhibitors
Source: PLoS One. 2014 Feb 18;9(2):e88908. doi: 10.1371/journal.pone.0088908 (PMC3928314; doi:10.1371/journal.pone.0088908)

**A**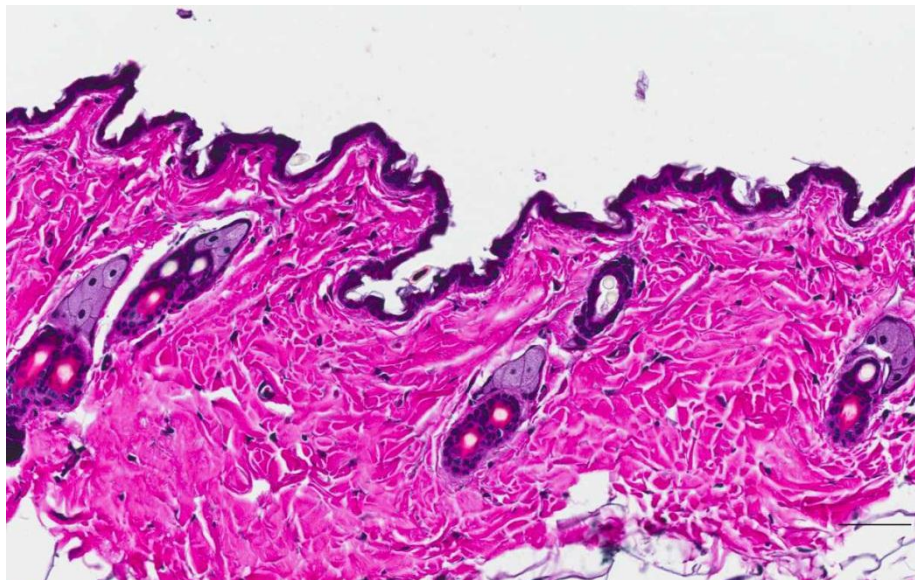

Vehicle, d14

**B**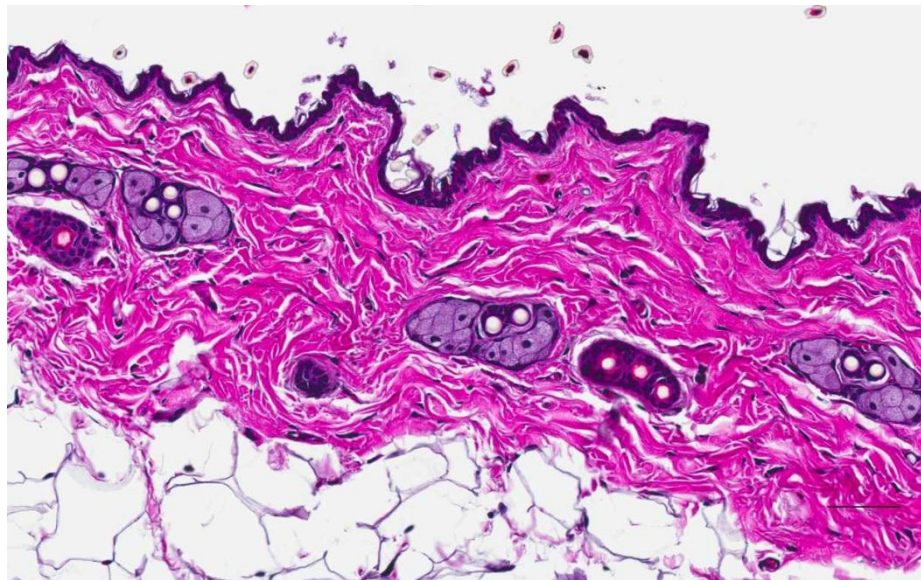

Cpd1, 3 mg/kg, d14 (Scoring: not remarkable)

**C**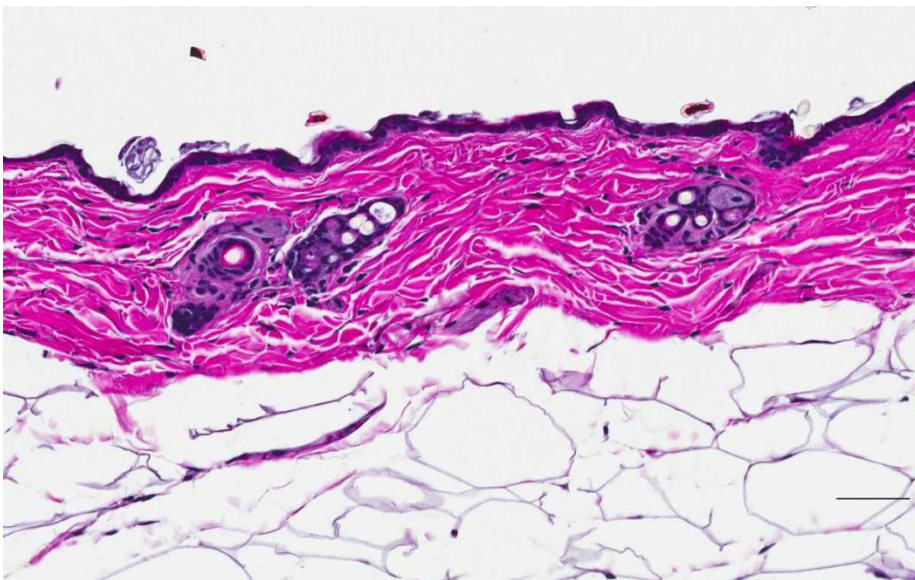

Cpd2, 30 mg/kg, d14 (Scoring: moderate to marked)

Supplement: Figure S1 — DGAT1 inhibitors with high lipophilicity induce sebaceous gland atrophy. Shown are hematoxylin and eosin stains of ventral skin biopsies from DIO mice treated with either vehicle (A), Cpd1 (B), or Cpd2 (C) for 14 days at doses indicated. Scoring refers to the histological adverse effect score as described in Table 1. Bar = 50 µm. (PDF) [file pone.0088908.s001.pdf]
